# Supplementary material for: Digital Health Technologies for Alzheimer’s Disease and Related Dementias: Initial Results from a Landscape Analysis and Community Collaborative Effort
Source: J Prev Alzheimers Dis. 2024 Jun 6;11(5):1480–9. doi: 10.14283/jpad.2024.103 (PMC11436391; doi:10.14283/jpad.2024.103)
Supplement: Supplementary file 1 — Search terms; technologies and citations identified [file 42414_2024_103_MOESM1_ESM.docx]

**Supplementary Material: Search terms; technologies and citations identified**

1. Search terms

| **Number** | **Theme/COIs** | **Terms** |
| --- | --- | --- |
| 1 | Performing activities of daily living | Activities of daily living; independent activities of daily living, Instrumental activities of daily living; Finances; scheduling; managing medicine; medication, dressing; eating/meals |
| 2 | Sleep health | Sleep onset latency; sleep quality; sleep waking; sleep duration; sleep staging; circadian; sleep disturbance; daytime sleepiness; sleep quality |
| 3 | Memory | Encoding; Episodic memory; learning; remembering; recollection |
| 4 | Language | Vocabulary; communication; fluency; speech; language; lexicon; lexical; linguistic; verbal fluency |
| 5 | Social connection/activities | time spent; distance; location; calendar; social; sms; social media; activities; |
| 6 | Oculomotor | Reaction time; eye movements; pupillometry |
| 7 | Cognition | Learning; skills; technological confidence; self-efficacy; digital literacy; attention; executive function; social cognition; app usage, cognitive function; cognitive dysfunction; cognitive impairment; cognitive decline; dementia |
| 8 | Life space mobility | Gait Function; walking; transitions; physical activity; driving; time spent; distance; location; navigation; Variability of activity; spatial awareness; balance |
| 9 | Essential bodily function | Eating; food; swallowing; dressing; showering; using the bathroom; continence |
| 10 | Emotions/behavioral issues | Depression; anxiety; emotion; emotional lability; anger; outbursts; emotional blunting, aggression; agitation; delirium; behavioral change; impulse control; impulsive; personality |
| 11 | Population of interest diagnostic status | Preclinical/MCI/Prodromal/Mild AD (Amnestic/non amnestic); Subjective Cognitive Decline; Subjective Cognitive Concerns; Cognitive Dysfunction; Mild Cognitive Impairment; Moderate AD; Severe AD; Vascular Dementia; FTD; Lewy body; General dementia; Mixed-Domain Cognitive Impairment; Mixed AD/Vascular Dementia |
|  | | |

1. Technologies and evidence captured through literature search & partner review

|  | **Model** | **Manufacturer** | **Evidence Value** |  |
| --- | --- | --- | --- | --- |
|  | Actiwatch Spectrum | Philips | Au-Yeung, W. T. M., Miller, L., Beattie, Z., May, R., Cray, H. V., Kabelac, Z., ... & Vahia, I. V. (2022). Monitoring behaviors of patients with late-stage dementia using passive environmental sensing approaches: a case series. The American Journal of Geriatric Psychiatry, 30(1), 1-11. https://doi.org/10.1016%2Fj.jagp.2021.04.008 |  |
|  | Actiwatch Spectrum | Philips | Au‐Yeung, W. T. M., Miller, L., Beattie, Z., Dodge, H. H., Reynolds, C., Vahia, I., & Kaye, J. (2020). Sensing a problem: Proof of concept for characterizing and predicting agitation. Alzheimer's & Dementia: Translational Research & Clinical Interventions, 6(1), e12079.Chicago https://doi.org/10.1002%2Ftrc2.12079 |  |
|  | AI Dialogue Agent | Experimental | Tang, F., Uchendu, I., Wang, F., Dodge, H. H., & Zhou, J. (2020). Scalable diagnostic screening of mild cognitive impairment using AI dialogue agent. Scientific reports, 10(1), 1-11. https://doi.org/10.1038/s41598-020-61994-0 |  |
|  | Altoida ADPS | Altoida | Rai, L., Boyle, R., Brosnan, L., Rice, H., Farina, F., Tarnanas, I., & Whelan, R. (2020). Digital biomarkers based individualized prognosis for people at risk of dementia: the AltoidaML Multi-site External validation Study. In GeNeDis 2018: Computational Biology and Bioinformatics (pp. 157-171). Springer International Publishing. https://doi.org/10.1007/978-3-030-32622-7_14 |  |
|  | Altoida DNS | Altoida | Harms, R. L., Ferrari, A., Meier, I. B., Martinkova, J., Santus, E., Marino, N., ... & Santuccione Chadha, A. (2022). Digital biomarkers and sex impacts in Alzheimer’s disease management—potential utility for innovative 3P medicine approach. EPMA Journal, 13(2), 299-313. https://doi.org/10.1007/s13167-022-00284-3 |  |
|  | Altoida DNS | Altoida | Meier, I. B., Buegler, M., Harms, R., Seixas, A., Çöltekin, A., & Tarnanas, I. (2021). Using a Digital Neuro Signature to measure longitudinal individual-level change in Alzheimer’s disease: The Altoida large cohort study. NPJ Digital Medicine, 4(1), 101. https://doi.org/10.1038/s41746-021-00470-z |  |
|  | Altoida DNS | Altoida | Muurling, M., de Boer, C., Kozak, R., Religa, D., Koychev, I., Verheij, H., ... & Visser, P. J. (2021). Remote monitoring technologies in Alzheimer’s disease: design of the RADAR-AD study. Alzheimer's research & therapy, 13(1), 1-13. https://doi.org/10.1186/s13195-021-00825-4 |  |
|  | Altoida DNS | Altoida | Seixas, A. A., Rajabli, F., Pericak-Vance, M. A., Jean-Louis, G., Harms, R. L., & Tarnanas, I. (2022). Associations of digital neuro-signatures with molecular and neuroimaging measures of brain resilience: The altoida large cohort study. Frontiers in psychiatry, 13, 899080. https://doi.org/10.3389%2Ffpsyt.2022.899080 |  |
|  | Altoida DNS | Altoida | Sood, M., Harms, R., Bügler, M., Tarnanas, I., Coello, N., & Fröhlich, H. (2021). Evaluating digital device technology in Alzheimer’s disease via artificial intelligence. Alzheimer's & Dementia, 17, e053574. https://doi.org/10.1002/alz.053574 |  |
|  | Altoida DNS | Altoida | Tarnanas, I., Vlamos, P., & RADAR-AD Consortium. (2021). Can detection and prediction models for Alzheimer’s Disease be applied to Prodromal Parkinson’s Disease using explainable artificial intelligence? A brief report on Digital Neuro Signatures. Open Research Europe, 1(146), 146. https://doi.org/10.12688/openreseurope.14216.2 |  |
|  | Altoida iADL task | Altoida | Tarnanas, I., Tsolaki, A., Wiederhold, M., Wiederhold, B., & Tsolaki, M. (2015). Five-year biomarker progression variability for Alzheimer's disease dementia prediction: Can a complex instrumental activities of daily living marker fill in the gaps?. Alzheimer's & Dementia: Diagnosis, Assessment & Disease Monitoring, 1(4), 521-532. https://doi.org/10.1016/j.dadm.2015.10.005 |  |
|  | Altoida Neuro Motor Index | Altoida | Buegler, M., Harms, R. L., Balasa, M., Meier, I. B., Exarchos, T., Rai, L., ... & Tarnanas, I. (2020). Digital biomarker‐based individualized prognosis for people at risk of dementia. Alzheimer's & Dementia: Diagnosis, Assessment & Disease Monitoring, 12(1), e12073. https://doi.org/10.1002%2Fdad2.12073 |  |
|  | Ambulatory Research in Cognition (ARC) app | Cognitive Technology Research Laboratory (CTRLab), Washington University in St. Louis; Sage Bionetworks | Nicosia, J., Aschenbrenner, A. J., Balota, D. A., Sliwinski, M. J., Tahan, M., Adams, S., ... & Hassenstab, J. (2022). Unsupervised high-frequency smartphone-based cognitive assessments are reliable, valid, and feasible in older adults at risk for Alzheimer’s disease. Journal of the International Neuropsychological Society, 1-13. https://doi.org/10.1017%2FS135561772200042X |  |
|  | Ambulatory Research in Cognition (ARC) app | Cognitive Technology Research Laboratory (CTRLab), Washington University in St. Louis; Sage Bionetworks | Wilks, H., Aschenbrenner, A. J., Gordon, B. A., Balota, D. A., Fagan, A. M., Musiek, E., ... & Hassenstab, J. (2021). Sharper in the morning: Cognitive time of day effects revealed with high-frequency smartphone testing. Journal of Clinical and Experimental Neuropsychology, 43(8), 825-837. https://doi.org/10.1080%2F13803395.2021.2009447 |  |
|  | Amsterdam IADL Questionnaire | Alzheimer Center VU University Medical Center, Amsterdam, The Netherlands | Muurling, M., de Boer, C., Kozak, R., Religa, D., Koychev, I., Verheij, H., ... & Visser, P. J. (2021). Remote monitoring technologies in Alzheimer’s disease: design of the RADAR-AD study. Alzheimer's research & therapy, 13(1), 1-13. https://doi.org/10.1186/s13195-021-00825-4 |  |
|  | Automatic Speech Recognition algorithm | Experimental | Tóth, L., Hoffmann, I., Gosztolya, G., Vincze, V., Szatlóczki, G., Bánréti, Z., ... & Kálmán, J. (2018). A speech recognition-based solution for the automatic detection of mild cognitive impairment from spontaneous speech. Current Alzheimer Research, 15(2), 130-138. https://doi.org/10.2174/1567205014666171121114930 |  |
|  | AX3 | Axivity | Corbi, A., & Burgos, D. (2022). Connection between sleeping patterns and cognitive deterioration in women with Alzheimer’s disease. Sleep and Breathing, 1-11. https://doi.org/10.1007/s11325-021-02327-x |  |
|  | AX3 | Axivity | Mc Ardle, R., Del Din, S., Donaghy, P., Galna, B., Thomas, A. J., & Rochester, L. (2021). The impact of environment on gait assessment: considerations from real-world gait analysis in dementia subtypes. Sensors, 21(3), 813. https://doi.org/10.3390/s21030813 |  |
|  | AX3 | Axivity | Mc Ardle, R., Del Din, S., Donaghy, P., Galna, B., Thomas, A., & Rochester, L. (2020). Factors that influence habitual activity in mild cognitive impairment and dementia. Gerontology, 66(2), 197-208. https://doi.org/10.1159/000502288 |  |
|  | AX3 | Axivity | Mc Ardle, R., Del Din, S., Galna, B., Thomas, A., & Rochester, L. (2020). Differentiating dementia disease subtypes with gait analysis: feasibility of wearable sensors?. Gait & posture, 76, 372-376. https://doi.org/10.1016/j.gaitpost.2019.12.028 |  |
|  | AX3 | Axivity | Muurling, M., de Boer, C., Kozak, R., Religa, D., Koychev, I., Verheij, H., ... & Visser, P. J. (2021). Remote monitoring technologies in Alzheimer’s disease: design of the RADAR-AD study. Alzheimer's research & therapy, 13(1), 1-13. https://doi.org/10.1186/s13195-021-00825-4 |  |
|  | AX3 | Axivity | Wang, J., Battioui, C., McCarthy, A., Dang, X., Zhang, H., Man, A., ... & Biglan, K. (2022). Evaluating the Use of Digital Biomarkers to Test Treatment Effects on Cognition and Movement in Patients with Lewy Body Dementia. Journal of Parkinson's Disease, (Preprint), 1-14.Chicago https://doi.org/10.3233/jpd-213126 |  |
|  | Bedside AS (BAS) Task | Experimental | Hellmuth, J., Mirsky, J., Heuer, H. W., Matlin, A., Jafari, A., Garbutt, S., ... & Boxer, A. L. (2012). Multicenter validation of a bedside antisaccade task as a measure of executive function. Neurology, 78(23), 1824-1831. https://doi.org/10.1212%2FWNL.0b013e318258f785 |  |
|  | Bittium Faros 180 | Bittium | Godkin, F. E., Turner, E., Demnati, Y., Vert, A., Roberts, A., Swartz, R. H., ... & Van Ooteghem, K. (2021). Feasibility of a continuous, multi-sensor remote health monitoring approach in persons living with neurodegenerative disease. Journal of neurology, 1-14. https://doi.org/10.1007/s00415-021-10831-z |  |
|  | Boston cognitive assessment (BOCA) | BellCurveAndMe | Vyshedskiy, A., Netson, R., Fridberg, E., Jagadeesan, P., Arnold, M., Barnett, S., ... & Gold, D. (2022). Boston cognitive assessment (BOCA)—a comprehensive self-administered smartphone-and computer-based at-home test for longitudinal tracking of cognitive performance. BMC neurology, 22(1), 1-12. https://doi.org/10.1186/s12883-022-02620-6 |  |
|  | Boston Remote Assessment for Neurocognitive Health (BRANCH) | Harvard Medical School / Mass General Brigham | Papp, K. V., Samaroo, A., Chou, H. C., Buckley, R., Schneider, O. R., Hsieh, S., ... & Amariglio, R. E. (2021). Unsupervised mobile cognitive testing for use in preclinical Alzheimer's disease. Alzheimer's & Dementia: Diagnosis, Assessment & Disease Monitoring, 13(1), e12243. https://doi.org/10.1002/dad2.12243 |  |
|  | Brain Health Assessment (BHA) | Cogniciti | Paterson, T. S., Sivajohan, B., Gardner, S., Binns, M. A., Stokes, K. A., Freedman, M., ... & Troyer, A. K. (2022). Accuracy of a self-administered online cognitive assessment in detecting amnestic mild cognitive impairment. The Journals of Gerontology: Series B, 77(2), 341-350. https://doi.org/10.1093/geronb/gbab097 |  |
|  | CANTAB | Cambridge Cognition | Baker, E., Dente, P., Backx, R., Lowther, M., Cotter, J., Cormack, F. K., & Barnett, J. (2020). Delivery of CANTAB assessments across diverse health systems: developing topics. Alzheimer's & Dementia, 16, e047550. https://doi.org/10.1002/alz.047550 |  |
|  | CANTAB | Cambridge Cognition | McWilliams, E. C., Barbey, F. M., Dyer, J. F., Islam, M. N., McGuinness, B., Murphy, B., ... & Buick, A. R. (2021). Feasibility of repeated assessment of cognitive function in older adults using a wireless, mobile, dry-EEG headset and tablet-based games. Frontiers in psychiatry, 12, 574482. https://doi.org/10.3389/fpsyt.2021.574482 |  |
|  | CANTAB | Cambridge Cognition | CC provided 140+ abstracts of clinical studies featuring CANTAB in Alzheimer's patient populations. Available upon request. |  |
|  | CANTAB | Cambridge Cognition | CC provided 72 abstracts featuring CANTAB in MCI patient populations. Available upon request. |  |
|  | CANTAB | Cambridge Cognition | CC provided abstracts featuring CANTAB in Lewy Body patient populations. Available upon request. |  |
|  | CANTAB | Cambridge Cognition | CC provided 12 abstracts featuring CANTAB in frontotemporal dementia populations. Available upon request. |  |
|  | CANTAB | Cambridge Cognition | CC provided 60+ abstracts of clinical studies featuring CANTAB in dementia populations. Available upon request. |  |
|  | Cascade Neural Network | Experimental | You, Z., Zeng, R., Lan, X., Ren, H., You, Z., Shi, X., ... & Hu, X. (2020). Alzheimer's disease classification with a cascade neural network. Frontiers in Public Health, 8, 584387. https://doi.org/10.3389/fpubh.2020.584387 |  |
|  | Cognionics 2-channel EEG headband | CGX Systems | Casciola, A. A., Carlucci, S. K., Kent, B. A., Punch, A. M., Muszynski, M. A., Zhou, D., ... & Nygaard, H. B. (2021). A deep learning strategy for automatic sleep staging based on two-channel EEG headband data. Sensors, 21(10), 3316. https://doi.org/10.3390/s21103316 |  |
|  | Cogstate Brief Battery | Cogstate | Maruff, P., Lim, Y. Y., Darby, D., Ellis, K. A., Pietrzak, R. H., Snyder, P. J., ... & Masters, C. L. (2013). Clinical utility of the cogstate brief battery in identifying cognitive impairment in mild cognitive impairment and Alzheimer’s disease. BMC psychology, 1(1), 1-11. https://doi.org/10.1186/2050-7283-1-30 |  |
|  | Computerized Cognitive Composite (C3) battery | Cogstate | Jutten, R. J., Rentz, D. M., Fu, J. F., Mayblyum, D. V., Amariglio, R. E., Buckley, R. F., ... & Papp, K. V. (2022). Monthly at-home computerized cognitive testing to detect diminished practice effects in preclinical Alzheimer's disease. Frontiers in Aging Neuroscience, 13, 800126.https://doi.org/10.3389/fnagi.2021.800126 |  |
|  | Cumulus Platform | Cumulus Neuroscience | 510(k) Clearance K221963 |  |
|  | Cumulus Platform | Cumulus Neuroscience | McWilliams, E. C., Barbey, F. M., Dyer, J. F., Islam, M. N., McGuinness, B., Murphy, B., ... & Buick, A. R. (2021). Feasibility of repeated assessment of cognitive function in older adults using a wireless, mobile, dry-EEG headset and tablet-based games. Frontiers in psychiatry, 12, 574482. https://doi.org/10.3389/fpsyt.2021.574482 |  |
|  | Cumulus Platform | Cumulus Neuroscience | UKCA Marking |  |
|  | Digital Clock and Recall (DCR) | Linus Health | Banks, R. E., Higgins, C., Bates, D., Gomes‐Osman, J. R., Pascual‐Leone, A., & Tobyne, S. E. (2022). Enhanced Machine Learning Classification of Cognitive Impairment with Multimodal Digital Biomarkers. Alzheimer's & Dementia, 18, e060485. https://doi.org/10.1002/alz.060485 |  |
|  | Digital Clock Drawing Test | Linus Health; Lahey Clinic and Massachusetts Institute of Technology | Binaco, R., Calzaretto, N., Epifano, J., McGuire, S., Umer, M., Emrani, S., ... & Polikar, R. (2020). Machine learning analysis of digital clock drawing test performance for differential classification of mild cognitive impairment subtypes versus Alzheimer’s disease. Journal of the International Neuropsychological Society, 26(7), 690-700. https://doi.org/10.1017/s1355617720000144 |  |
|  | Discrete Tension Indicator (DTI-2) | Philips | Melander, C. A., Kikhia, B., Olsson, M., Wälivaara, B. M., & Sävenstedt, S. (2019). The impact of using measurements of electrodermal activity in the assessment of problematic behaviour in dementia. Dementia and Geriatric Cognitive Disorders Extra, 8(3), 333-347. https://doi.org/10.1159/000493339 |  |
|  | Dreem 3 headband | Beacon | Wilson, S., Ardle, R. M., Tolley, C., & Slight, S. (2022). Usability and acceptability of wearable technology in the early detection of dementia. Alzheimer's & Dementia, 18, e059820. https://doi.org/10.1002/alz.059820 |  |
|  | DryBuddy | DryBuddy Enuresis Solutions | Rose, K. M., Lach, J., Perkhounkova, Y., Gong, J., Dandu, S. R., Dickerson, R., ... & Stankovic, J. (2018). Use of body sensors to examine nocturnal agitation, sleep, and urinary incontinence in individuals with Alzheimer's disease. Journal of Gerontological Nursing, 44(8), 19-26. https://doi.org/10.3928/00989134-20180626-03 |  |
|  | DynaPort Hybrid | McRoberts | Van Lummel, R. C., Walgaard, S., Hobert, M. A., Maetzler, W., Van Dieën, J. H., Galindo-Garre, F., & Terwee, C. B. (2016). Intra-rater, inter-rater and test-retest reliability of an instrumented timed up and go (iTUG) test in patients with Parkinson’s disease. PloS one, 11(3), e0151881. https://doi.org/10.1371/journal.pone.0151881 |  |
|  | DynaPort Hybrid | McRoberts | Van Uem, J. M., Walgaard, S., Ainsworth, E., Hasmann, S. E., Heger, T., Nussbaum, S., ... & Maetzler, W. (2016). Quantitative timed-up-and-go parameters in relation to cognitive parameters and health-related quality of life in mild-to-moderate Parkinson's disease. PloS one, 11(4), e0151997. https://doi.org/10.1371%2Fjournal.pone.0151997 |  |
|  | Dynaport MoveMonitor | McRoberts | Schootemeijer, S., Weijer, R. H., Hoozemans, M. J., van Schooten, K. S., Delbaere, K., & Pijnappels, M. (2020). Association between daily-life gait quality characteristics and physiological fall risk in older people. Sensors, 20(19), 5580. https://doi.org/10.3390%2Fs20195580 |  |
|  | DynaPort MT | McRoberts | Buchman, A. S., Dawe, R. J., Leurgans, S. E., Curran, T. A., Truty, T., Yu, L., ... & Bennett, D. A. (2020). Different combinations of mobility metrics derived from a wearable sensor are associated with distinct health outcomes in older adults. The Journals of Gerontology: Series A, 75(6), 1176-1183. https://doi.org/10.1093%2Fgerona%2Fglz160 |  |
|  | Emerald | Emerald Innovations | Au-Yeung, W. T. M., Miller, L., Beattie, Z., May, R., Cray, H. V., Kabelac, Z., ... & Vahia, I. V. (2022). Monitoring behaviors of patients with late-stage dementia using passive environmental sensing approaches: a case series. The American Journal of Geriatric Psychiatry, 30(1), 1-11. https://doi.org/10.1016/j.jagp.2021.04.008 |  |
|  | Emerald | Emerald Innovations | Vahia, I. V., Kabelac, Z., Hsu, C. Y., Forester, B. P., Monette, P., May, R., ... & Katabi, D. (2020). Radio signal sensing and signal processing to monitor behavioral symptoms in dementia: a case study. The American Journal of Geriatric Psychiatry, 28(8), 820-825. https://doi.org/10.1016/j.jagp.2020.02.012 |  |
|  | Emfit Sleep Sensor | Emfit | Au‐Yeung, W. T. M., Miller, L., Beattie, Z., Dodge, H. H., Reynolds, C., Vahia, I., & Kaye, J. (2020). Sensing a problem: Proof of concept for characterizing and predicting agitation. Alzheimer's & Dementia: Translational Research & Clinical Interventions, 6(1), e12079. https://doi.org/10.1002%2Ftrc2.12079 |  |
|  | Convolutional neural network using mobility data | Experimental | Bringas, S., Salomón, S., Duque, R., Lage, C., & Montaña, J. L. (2020). Alzheimer’s disease stage identification using deep learning models. Journal of Biomedical Informatics, 109, 103514. https://doi.org/10.1016/j.jbi.2020.103514 |  |
|  | Convolutional neural network for gaze location estimation | Experimental | Jiang, Z., Seyedi, S., Haque, R. U., Pongos, A. L., Vickers, K. L., Manzanares, C. M., ... & Clifford, G. D. (2022). Automated analysis of facial emotions in subjects with cognitive impairment. Plos one, 17(1), e0262527. https://doi.org/10.1371/journal.pone.0262527 |  |
|  | Convolutional neural network + LSTM for voice analysis | Experimental | Xue, C., Karjadi, C., Paschalidis, I. C., Au, R., & Kolachalama, V. B. (2021). Detection of dementia on voice recordings using deep learning: a Framingham Heart Study. Alzheimer's Research & Therapy, 13, 1-15. https://doi.org/10.1186/s13195-021-00888-3 |  |
|  | EyeLink 1000 Plus | SR Research | Pavisic, I. M., Pertzov, Y., Nicholas, J. M., O’Connor, A., Lu, K., Yong, K. X., ... & Crutch, S. J. (2021). Eye-tracking indices of impaired encoding of visual short-term memory in familial Alzheimer’s disease. Scientific reports, 11(1), 8696. https://doi.org/10.1038/s41598-021-88001-4 |  |
|  | EyeLink 1000 Plus | SR Research | Russell, L. L., Greaves, C. V., Convery, R. S., Nicholas, J., Warren, J. D., Kaski, D., & Rohrer, J. D. (2021). Novel instructionless eye tracking tasks identify emotion recognition deficits in frontotemporal dementia. Alzheimer's research & therapy, 13, 1-11. https://doi.org/10.1186/s13195-021-00775-x |  |
|  | FallSkip | Biomechanical Institute of Valencia, Valencia, Spain | Pedrero-Sánchez, J. F., Belda-Lois, J. M., Serra-Ano, P., Ingles, M., & Lopez-Pascual, J. (2022). Classification of healthy, Alzheimer and Parkinson populations with a multi-branch neural network. Biomedical Signal Processing and Control, 75, 103617. https://doi.org/10.1016/j.bspc.2022.103617 |  |
|  | FallSkip | Biomechanical Institute of Valencia, Valencia, Spain | Serra-Añó, P., Pedrero-Sánchez, J. F., Hurtado-Abellán, J., Inglés, M., Espí-López, G. V., & López-Pascual, J. (2019). Mobility assessment in people with Alzheimer disease using smartphone sensors. Journal of NeuroEngineering and Rehabilitation, 16, 1-9. https://doi.org/10.1186/s12984-019-0576-y |  |
|  | Fitbit Charge 3 | Fitbit | Muurling, M., de Boer, C., Kozak, R., Religa, D., Koychev, I., Verheij, H., ... & Visser, P. J. (2021). Remote monitoring technologies in Alzheimer’s disease: design of the RADAR-AD study. Alzheimer's research & therapy, 13(1), 1-13. https://doi.org/10.1186/s13195-021-00825-4 |  |
|  | Fitbit Charge 4 | Fitbit | Wilson, S., Ardle, R. M., Tolley, C., & Slight, S. (2022). Usability and acceptability of wearable technology in the early detection of dementia. Alzheimer's & Dementia, 18, e059820. https://doi.org/10.1002/alz.059820 |  |
|  | G2 Tracking Device | Azuga | Bayat, S., Babulal, G. M., Schindler, S. E., Fagan, A. M., Morris, J. C., Mihailidis, A., & Roe, C. M. (2021). GPS driving: a digital biomarker for preclinical Alzheimer disease. Alzheimer's Research & Therapy, 13(1), 1-9. https://doi.org/10.1186/s13195-021-00852-1 |  |
|  | GAITRite | CIR Systems | Mc Ardle, R., Del Din, S., Galna, B., Thomas, A., & Rochester, L. (2020). Differentiating dementia disease subtypes with gait analysis: feasibility of wearable sensors?. Gait & posture, 76, 372-376. https://doi.org/10.1016/j.gaitpost.2019.12.028 |  |
|  | GAITRite | CIR Systems | Tung, J. Y., Rose, R. V., Gammada, E., Lam, I., Roy, E. A., Black, S. E., & Poupart, P. (2014). Measuring life space in older adults with mild-to-moderate Alzheimer's disease using mobile phone GPS. Gerontology, 60(2), 154-162. https://doi.org/10.1159/000355669 |  |
|  | Garmin vívosmart HR | Garmin | Svetnik, V., Wang, T. C., Ceesay, P., Snyder, E., Ceren, O., Bliwise, D., ... & Herring, W. J. (2021). Pilot evaluation of a consumer wearable device to assess sleep in a clinical polysomnography trial of suvorexant for treating insomnia in patients with Alzheimer's disease. Journal of Sleep Research, 30(6), e13328. https://doi.org/10.1111/jsr.13328 |  |
|  | GazePoint eye tracker | Gazepoint | Parra, M. A., Granada, J., & Fernández, G. (2022). Memory‐driven eye movements prospectively predict dementia in people at risk of Alzheimer's disease. Alzheimer's & Dementia: Diagnosis, Assessment & Disease Monitoring, 14(1), e12386. https://doi.org/10.1002/dad2.12386 |  |
|  | GENEActiv | Activinsights | Godkin, F. E., Turner, E., Demnati, Y., Vert, A., Roberts, A., Swartz, R. H., ... & Van Ooteghem, K. (2021). Feasibility of a continuous, multi-sensor remote health monitoring approach in persons living with neurodegenerative disease. Journal of neurology, 1-14. https://doi.org/10.1007/s00415-021-10831-z |  |
|  | GT3x+ | ActiGraph | Ghosal, R., Varma, V. R., Volfson, D., Urbanek, J., Hausdorff, J. M., Watts, A., & Zipunnikov, V. (2022). Scalar on time-by-distribution regression and its application for modelling associations between daily-living physical activity and cognitive functions in Alzheimer’s Disease. Scientific reports, 12(1), 11558. https://doi.org/10.1038/s41598-022-15528-5 |  |
|  | JiBuEn gait analysis system | Experimental | Huang, S., Zhou, X., Liu, Y., Luo, J., Lv, Z., Shang, P., ... & Xie, H. (2022). High Fall Risk Associated With Memory Deficit and Brain Lobes Atrophy Among Elderly With Amnestic Mild Cognitive Impairment and Mild Alzheimer’s Disease. Frontiers in Neuroscience, 16, 793. https://doi.org/10.3389/fnins.2022.896437 |  |
|  | ki:e SB-C | ki elements | König, A., Linz, N., Baykara, E., Tröger, J., Ritchie, C., Saunders, S., ... & Hansson, O. (2023). Screening over Speech in Unselected Populations for Clinical Trials in AD (PROSPECT-AD): Study Design and Protocol. The Journal of Prevention of Alzheimer's Disease, 1-8. https://doi.org/10.14283/jpad.2023.11 |  |
|  | ki:e SB-C | ki elements | Linz, N., Mallick, E., Mina, M., Possemis, N., König, A., Huurne, D. B. T., ... & Tröger, J. (2022). validation of the ki: e SB‐C a Novel Digital Speech Biomarker for Cognition in a Dutch Memory Clinic Population. [Poster presentation]. Alzheimer's & Dementia, 18, e061600. |  |
|  | ki:e SB-C | ki elements | Ruhmel, S., Troger, J., Linz, N., Herrmann, J., Quiceno, M., & Langel, K. (n.d.) Pre-Screening Prodromal AD Trial Populations over the Telephone Using a Speech Biomarker for Cognition — Preliminary Results from AUTONOMY Phase 2 AD Trial Recruitment. [Poster presentation]. Janssen Research & Development, LLC, United States. ki:elements, Saarbrücken, Germany. Janssen-Cilag, Spain. |  |
|  | ki:e SB-C | ki elements | Gregory S, Linz N, König A, Langel K, Pullen H, Luz S, Harrison J, Ritchie CW. Remote data collection speech analysis and prediction of the identification of Alzheimer’s disease biomarkers in people at risk for Alzheimer’s disease dementia: the Speech on the Phone Assessment (SPeAk) prospective observational study protocol. BMJ open. 2022 Mar 1;12(3):e052250. https://doi.org/10.1136%2Fbmjopen-2021-052250 |  |
|  | ki:e SB-C | ki elements | Schäfer S, Mallick E, Schwed L, König A, Zhao J, Linz N, Bodin TH, Skoog J, Possemis N, Ter Huurne D, Zettergren A. Screening for mild cognitive impairment using a machine learning classifier and the remote speech biomarker for cognition: evidence from two clinically relevant cohorts. Journal of Alzheimer's Disease. 2023 Jan 1;91(3):1165-71. https://doi.org/10.3233%2FJAD-220762 |  |
|  | ki:e SB-C | ki elements | Ter Huurne, D., Ramakers, I., Possemis, N., Banning, L., Gruters, A., Van Asbroeck, S., ... & de Vugt, M. (2023). The Accuracy of Speech and Linguistic Analysis in Early Diagnostics of Neurocognitive Disorders in a Memory Clinic Setting. Archives of Clinical Neuropsychology, acac105. https://doi.org/10.1093/arclin/acac105 |  |
|  | ki:e SB-C | ki elements | Tröger, J., Baykara, E., Zhao, J., Ter Huurne, D., Possemis, N., Mallick, E., ... & Ritchie, C. (2022). validation of the remote automated ki: e speech biomarker for cognition in mild cognitive impairment: Verification and validation following DiME V3 Framework. Digital biomarkers, 6(3), 107-116. https://doi.org/10.1159%2F000526471 |  |
|  | Kinect | Microsoft | König, A., Crispim-Junior, C. F., Covella, A. G. U., Bremond, F., Derreumaux, A., Bensadoun, G., ... & Robert, P. (2015). Ecological assessment of autonomy in instrumental activities of daily living in dementia patients by the means of an automatic video monitoring system. Frontiers in aging neuroscience, 7, 98. https://doi.org/10.3389/fnagi.2015.00098 |  |
|  | Kinect | Microsoft | Yang, Y. H., Lee, Y. H., Yen, C. W., Huang, L. C., Chang, Y. P., & Chien, C. F. (2022). Association between Cerebral Coordination Functions and Clinical Outcomes of Alzheimer’s Dementia. Brain Sciences, 12(10), 1370. https://doi.org/10.3390%2Fbrainsci12101370 |  |
|  | Kinect | Microsoft | You, Z., Zeng, R., Lan, X., Ren, H., You, Z., Shi, X., ... & Hu, X. (2020). Alzheimer's disease classification with a cascade neural network. Frontiers in Public Health, 8, 584387.Chicago https://doi.org/10.3389/fpubh.2020.584387 |  |
|  | Kinesis Balance | Linus Health Europe | Greene, B. R., Doheny, E. P., McManus, K., & Caulfield, B. (2022). Estimating balance, cognitive function, and falls risk using wearable sensors and the sit-to-stand test. Wearable technologies, 3, e9. https://doi.org/10.1017/wtc.2022.6 |  |
|  | Kinesis Gait | Linus Health Europe | Ader, L. G. M., Greene, B. R., McManus, K., & Caulfield, B. (2021). Reliability of inertial sensor based spatiotemporal gait parameters for short walking bouts in community dwelling older adults. Gait & Posture, 85, 1-6. https://doi.org/10.1016/j.gaitpost.2021.01.010 |  |
|  | Kinesis QTUG | Linus Health Europe | Greene, B. R., McManus, K., Redmond, S. J., Caulfield, B., & Quinn, C. C. (2019). Digital assessment of falls risk, frailty, and mobility impairment using wearable sensors. NPJ digital medicine, 2(1), 125.https://doi.org/10.1038%2Fs41746-019-0204-z |  |
|  | LEGSys | BioSensics | Zhou, H., Lee, H., Lee, J., Schwenk, M., & Najafi, B. (2018). Motor planning error: toward measuring cognitive frailty in older adults using wearables. Sensors, 18(3), 926. https://doi.org/10.3390%2Fs18030926 |  |
|  | LEGSys | BioSensics | Zhou, H., Park, C., Shahbazi, M., York, M. K., Kunik, M. E., Naik, A. D., & Najafi, B. (2022). Digital biomarkers of cognitive frailty: The value of detailed gait assessment beyond gait speed. Gerontology, 68(2), 224-233. https://doi.org/10.1159%2F000515939 |  |
|  | LEGSys | BioSensics | Zhou, H., Sabbagh, M., Wyman, R., Liebsack, C., Kunik, M. E., & Najafi, B. (2017). Instrumented trail-making task to differentiate persons with no cognitive impairment, amnestic mild cognitive impairment, and Alzheimer disease: A proof of concept study. Gerontology, 63(2), 189-200.Chicago. https://doi.org/10.1159%2F000452309 |  |
|  | M2C2: Mobile Monitoring of Cognitive Change | National Institute on Aging's Mobile Toolbox | Thompson, L. I., Harrington, K. D., Roque, N., Strenger, J., Correia, S., Jones, R. N., ... & Sliwinski, M. J. (2022). A highly feasible, reliable, and fully remote protocol for mobile app‐based cognitive assessment in cognitively healthy older adults. Alzheimer's & Dementia: Diagnosis, Assessment & Disease Monitoring, 14(1), e12283. https://doi.org/10.1002/dad2.12283 |  |
|  | MetawearRG | MbientLab | Vahia, I. V., Kabelac, Z., Hsu, C. Y., Forester, B. P., Monette, P., May, R., ... & Katabi, D. (2020). Radio signal sensing and signal processing to monitor behavioral symptoms in dementia: a case study. The American Journal of Geriatric Psychiatry, 28(8), 820-825. https://doi.org/10.1016/j.jagp.2020.02.012 |  |
|  | Mezurio | Digital Phenotyping Laboratory, Oxford University Big Data Institute | Lancaster, C., Koychev, I., Blane, J., Chinner, A., Chatham, C., Taylor, K., & Hinds, C. (2020). Gallery Game: Smartphone-based assessment of long-term memory in adults at risk of Alzheimer’s disease. Journal of clinical and experimental neuropsychology, 42(4), 329-343. https://doi.org/10.1080/13803395.2020.1714551 |  |
|  | Mezurio | Digital Phenotyping Laboratory, Oxford University Big Data Institute | Lancaster, C., Koychev, I., Blane, J., Chinner, A., Wolters, L., & Hinds, C. (2020). Evaluating the feasibility of frequent cognitive assessment using the Mezurio smartphone app: observational and interview study in adults with elevated dementia risk. JMIR mHealth and uHealth, 8(4), e16142. https://doi.org/10.2196%2F16142 |  |
|  | Mezurio | Digital Phenotyping Laboratory, Oxford University Big Data Institute | Muurling, M., de Boer, C., Kozak, R., Religa, D., Koychev, I., Verheij, H., ... & Visser, P. J. (2021). Remote monitoring technologies in Alzheimer’s disease: design of the RADAR-AD study. Alzheimer's research & therapy, 13(1), 1-13. https://doi.org/10.1186/s13195-021-00825-4 |  |
|  | Mezurio | Digital Phenotyping Laboratory, Oxford University Big Data Institute | Wilson, S., Ardle, R. M., Tolley, C., & Slight, S. (2022). Usability and acceptability of wearable technology in the early detection of dementia. Alzheimer's & Dementia, 18, e059820. https://doi.org/10.1002/alz.059820 |  |
|  | mindLAMP | Dr. John Torous; Division of Digital Psychiatry at BIDMC | Weizenbaum, E. L., Fulford, D., Torous, J., Pinsky, E., Kolachalama, V. B., & Cronin-Golomb, A. (2022). Smartphone-based neuropsychological assessment in Parkinson’s disease: feasibility, validity, and contextually driven variability in cognition. Journal of the International Neuropsychological Society, 28(4), 401-413. https://doi.org/10.1017%2FS1355617721000503 |  |
|  | MindWave Mobile EEG | NeuroSky | Iliadou, P., Paliokas, I., Zygouris, S., Lazarou, E., Votis, K., Tzovaras, D., & Tsolaki, M. (2021). A comparison of traditional and serious game-based digital markers of cognition in older adults with mild cognitive impairment and healthy controls. Journal of Alzheimer's disease, 79(4), 1747-1759. https://doi.org/10.3233/jad-201300 |  |
|  | Miro Health Mobile Assessment Platform | MiroHealth | Sloane, K. L., Mefford, J. A., Zhao, Z., Xu, M., Zhou, G., Fabian, R., ... & Glenn, S. (2022). validation of a Mobile, Sensor-based Neurobehavioral Assessment With Digital Signal Processing and Machine-learning Analytics. Cognitive and Behavioral Neurology, 35(3), 169-178. https://doi.org/10.1097/wnn.0000000000000308 |  |
|  | mobile Detection Test of Language impairments in Adult (DTLA) | Experimental | Plonka, A., Mouton, A., Macoir, J., Tran, T. M., Derremaux, A., Robert, P., ... & Gros, A. (2021). Primary progressive aphasia: Use of graphical markers for an early and differential diagnosis. Brain Sciences, 11(9), 1198. https://doi.org/10.3390%2Fbrainsci11091198 |  |
|  | Motionlogger Actigraphs | Ambulatory Monitoring | Ancoli-Israel, S., Gehrman, P., Martin, J. L., Shochat, T., Marler, M., Corey-Bloom, J., & Levi, L. (2003). Increased light exposure consolidates sleep and strengthens circadian rhythms in severe Alzheimer's disease patients. Behavioral sleep medicine, 1(1), 22-36. https://doi.org/10.1207/s15402010bsm0101_4 |  |
|  | Motionlogger Actigraphs | Ambulatory Monitoring | Nagels, G., Engelborghs, S., Vloeberghs, E., Van Dam, D., Pickut, B. A., & De Deyn, P. P. (2006). Actigraphic measurement of agitated behaviour in dementia. International journal of geriatric psychiatry, 21(4), 388-393.https://doi.org/10.1002/gps.1483 |  |
|  | Motionlogger Actigraphs | Ambulatory Monitoring | Tranah, G. J., Blackwell, T., Stone, K. L., Ancoli‐Israel, S., Paudel, M. L., Ensrud, K. E., ... & SOF Research Group. (2011). Circadian activity rhythms and risk of incident dementia and mild cognitive impairment in older women. Annals of neurology, 70(5), 722-732. https://doi.org/10.1002/ana.22468 |  |
|  | move II Activity Sensor | movisens | Schaat, S., Koldrack, P., Yordanova, K., Kirste, T., & Teipel, S. (2020). Real-time detection of spatial disorientation in persons with mild cognitive impairment and dementia. Gerontology, 66(1), 85-94. https://doi.org/10.1159/000500971 |  |
|  | mSTS-MCI | Experimental | Park, J. H., Jung, M., Kim, J., Park, H. Y., Kim, J. R., & Park, J. H. (2018). Validity of a novel computerized screening test system for mild cognitive impairment. International psychogeriatrics, 30(10), 1455-1463. https://doi.org/10.1017/s1041610218000923 |  |
|  | NAIHA Neuro Cognitive Test (NNCT) | Experimental | Oliva, I., & Losa, J. (2022). validation of the Computerized Cognitive Assessment Test: NNCT. International Journal of Environmental Research and Public Health, 19(17), 10495. https://doi.org/10.3390/ijerph191710495 |  |
|  | Nemuri SCAN sensor | Paramount Bed | Fukuda, C., Higami, Y., Shigenobu, K., Kanemoto, H., & Yamakawa, M. (2022). Using a Non-Wearable Actigraphy in Nursing Care for Dementia With Lewy Bodies. American Journal of Alzheimer's Disease & Other Dementias®, 37, 15333175221082747. https://doi.org/10.1177/15333175221082747 |  |
|  | Nemuri SCAN sensor | Paramount Bed | Higami, Y., Yamakawa, M., Shigenobu, K., Kamide, K., & Makimoto, K. (2019). High frequency of getting out of bed in patients with Alzheimer's disease monitored by non‐wearable actigraphy. Geriatrics & Gerontology International, 19(2), 130-134. https://doi.org/10.1111/ggi.13565 |  |
|  | neotiv | neotiv | Berron D, Glanz W, Clark L, Basche K, Grande X, Güsten J, Billette OV, Hempen I, Naveed MH, Diersch N, Butryn M. A remote digital memory composite to detect cognitive impairment in memory clinic samples in unsupervised settings using mobile devices. npj Digital Medicine. 2024 Mar 26;7(1):79. https://doi.org/10.1038/s41746-024-00999-9 |  |
|  | neotiv | neotiv | Berron, D., Ziegler, G., Vieweg, P., Billette, O., Güsten, J., Grande, X., ... & Düzel, E. (2022). Feasibility of digital memory assessments in an unsupervised and remote study setting. Frontiers in digital health, 4, 892997. https://doi.org/10.3389/fdgth.2022.892997 |  |
|  | neotiv | neotiv | Öhman, F., Berron, D., Papp, K. V., Kern, S., Skoog, J., Hadarsson Bodin, T., ... & Schöll, M. (2022). Unsupervised mobile app-based cognitive testing in a population-based study of older adults born 1944. Frontiers in Digital Health, 4, 227. https://doi.org/10.3389/fdgth.2022.933265 |  |
|  | neotiv | neotiv | Regulatory certificate and available languages: see supplementary resources field |  |
|  | Neural Impairment Test Suite | Experimental | Lauraitis, A., Maskeliūnas, R., Damaševičius, R., & Krilavičius, T. (2020). A mobile application for smart computer-aided self-administered testing of cognition, speech, and motor impairment. Sensors, 20(11), 3236. https://doi.org/10.3390/s20113236 |  |
|  | Neurovocalix | Cambridge Cognition | Kaula, A. J., Cormack, F. K., & Taptiklis, N. (2021). Feasibility of repeated administration of automated verbal‐paired‐associate memory in older adults. Alzheimer's & Dementia, 17, e057804. https://doi.org/10.1002/alz.057804 |  |
|  | NIHTB-CB iPad English version | NIH | Ma, Y., Carlsson, C. M., Wahoske, M. L., Blazel, H. M., Chappell, R. J., Johnson, S. C., ... & Gleason, C. E. (2021). Latent factor structure and measurement invariance of the NIH toolbox cognition battery in an Alzheimer’s Disease research sample. Journal of the International Neuropsychological Society, 27(5), 412-425. https://doi.org/10.1017/s1355617720000922 |  |
|  | NYCE Ambient sensors | NYCE Sensors | Au‐Yeung, W. T. M., Miller, L., Beattie, Z., Dodge, H. H., Reynolds, C., Vahia, I., & Kaye, J. (2020). Sensing a problem: Proof of concept for characterizing and predicting agitation. Alzheimer's & Dementia: Translational Research & Clinical Interventions, 6(1), e12079. https://doi.org/10.1002%2Ftrc2.12079 |  |
|  | Opal | APDM Wearable Technologies | Kamil, R. J., Bakar, D., Ehrenburg, M., Wei, E. X., Pletnikova, A., Xiao, G., ... & Agrawal, Y. (2021). Detection of wandering behaviors using a body-worn inertial sensor in patients with cognitive impairment: A feasibility study. Frontiers in Neurology, 12, 529661.Chicago https://doi.org/10.3389/fneur.2021.529661 |  |
|  | OptiTrack | OptiTrack | Zhang, J., Xiao, Y., Li, Z. M., Wei, N., Lin, L., & Li, K. (2022). Reach-to-grasp kinematics and kinetics with and without visual feedback in early-stage Alzheimer’s disease. Journal of NeuroEngineering and Rehabilitation, 19(1), 1-15. https://doi.org/10.1186/s12984-022-01108-1 |  |
|  | ORCATECH Technology Platform | OHSU ORegon Center for Aging and TECHnology | Akl, A., Snoek, J., & Mihailidis, A. (2014, August). Generalized linear models of home activity for automatic detection of mild cognitive impairment in older adults. In 2014 36th Annual International Conference of the IEEE Engineering in Medicine and Biology Society (pp. 680-683). IEEE. https://doi.org/10.1109/embc.2014.6943682 |  |
|  | ORCATECH Technology Platform | OHSU ORegon Center for Aging and TECHnology | Akl, A., Taati, B., & Mihailidis, A. (2015). Autonomous unobtrusive detection of mild cognitive impairment in older adults. IEEE transactions on biomedical engineering, 62(5), 1383-1394. https://doi.org/10.1109/tbme.2015.2389149 |  |
|  | ORCATECH Technology Platform | OHSU ORegon Center for Aging and TECHnology | Au-Yeung, W. T. M., Miller, L., Beattie, Z., May, R., Cray, H. V., Kabelac, Z., ... & Vahia, I. V. (2022). Monitoring behaviors of patients with late-stage dementia using passive environmental sensing approaches: a case series. The American Journal of Geriatric Psychiatry, 30(1), 1-11. https://doi.org/10.1016/j.jagp.2021.04.008 |  |
|  | ORCATECH Technology Platform | OHSU ORegon Center for Aging and TECHnology | Au‐Yeung, W. T. M., Miller, L., Beattie, Z., Dodge, H. H., Reynolds, C., Vahia, I., & Kaye, J. (2020). Sensing a problem: Proof of concept for characterizing and predicting agitation. Alzheimer's & Dementia: Translational Research & Clinical Interventions, 6(1), e12079. https://doi.org/10.1002%2Ftrc2.12079 |  |
|  | ORCATECH Technology Platform | OHSU ORegon Center for Aging and TECHnology | Khan, T., & Jacobs, P. G. (2020). Prediction of mild cognitive impairment using movement complexity. IEEE journal of biomedical and health informatics, 25(1), 227-236. https://doi.org/10.1109/jbhi.2020.2985907 |  |
|  | ORCATECH Technology Platform | OHSU ORegon Center for Aging and TECHnology | Wu, C. Y., Beattie, Z., Mattek, N., Sharma, N., Kaye, J., & Dodge, H. H. (2021). Reproducibility and replicability of high‐frequency, in‐home digital biomarkers in reducing sample sizes for clinical trials. Alzheimer's & Dementia: Translational Research & Clinical Interventions, 7(1), e12220. https://doi.org/10.1002/trc2.12220 |  |
|  | PAMSys | BioSensics LLC | Freytag, J., Mishra, R. K., Street Jr, R. L., Catic, A., Dindo, L., Kiefer, L., ... & Naik, A. D. (2022). Using Wearable Sensors to Measure Goal Achievement in Older Veterans with Dementia. Sensors, 22(24), 9923. https://doi.org/10.3390/s22249923 |  |
|  | Passive Infrared (PIR) Sensors | DomoSafety SA | Botros, A. A., Schuetz, N., Röcke, C., Weibel, R., Martin, M., Müri, R. M., & Nef, T. (2022). Eigenbehaviour as an Indicator of Cognitive Abilities. Sensors, 22(7), 2769. https://doi.org/10.3390/s22072769 |  |
|  | Physilog | MindMaze | Kuan, Y. C., Huang, L. K., Wang, Y. H., Hu, C. J., Tseng, I. J., Chen, H. C., & Lin, L. F. (2021). Balance and gait performance in older adults with early-stage cognitive impairment. Eur. J. Phys. Rehabil. Med, 57, 560-567. https://doi.org/10.23736/s1973-9087.20.06550-8 |  |
|  | Praat | Paul Boersma, David Weenink at University of Amsterdam | del Carmen Pérez-Sánchez, M., González-Nosti, M., Cuetos, F., Martínez, C., & Álvarez-Cañizo, M. (2021). Reading fluency in Spanish patients with Alzheimer’s disease. Current Alzheimer Research, 18(3), 243-255. https://doi.org/10.2174/1567205018666210608102012 |  |
|  | Praat | Paul Boersma, David Weenink at University of Amsterdam | López-de-Ipiña, K., Alonso, J. B., Travieso, C. M., Solé-Casals, J., Egiraun, H., Faundez-Zanuy, M., ... & de Lizardui, U. M. (2013). On the selection of non-invasive methods based on speech analysis oriented to automatic Alzheimer disease diagnosis. Sensors, 13(5), 6730-6745. https://doi.org/10.3390/s130506730 |  |
|  | Praat | Paul Boersma, David Weenink at University of Amsterdam | Tóth, L., Hoffmann, I., Gosztolya, G., Vincze, V., Szatlóczki, G., Bánréti, Z., ... & Kálmán, J. (2018). A speech recognition-based solution for the automatic detection of mild cognitive impairment from spontaneous speech. Current Alzheimer Research, 15(2), 130-138. https://doi.org/10.2174/1567205014666171121114930 |  |
|  | Praat | Paul Boersma, David Weenink at University of Amsterdam | Vincze, V., Szatlóczki, G., Tóth, L., Gosztolya, G., Pákáski, M., Hoffmann, I., & Kálmán, J. (2021). Telltale silence: temporal speech parameters discriminate between prodromal dementia and mild Alzheimer’s disease. Clinical Linguistics & Phonetics, 35(8), 727-742. https://doi.org/10.1080/02699206.2020.1827043 |  |
|  | Pro Pod 5 | Trackershop | Ghosh, A., Puthusseryppady, V., Chan, D., Mascolo, C., & Hornberger, M. (2022). Machine learning detects altered spatial navigation features in outdoor behaviour of Alzheimer’s disease patients. Scientific Reports, 12(1), 3160. https://doi.org/10.1038/s41598-022-06899-w |  |
|  | Qualisys ProReflex | Qualisys | Ansai, J. H., de Andrade, L. P., Rossi, P. G., Nakagawa, T. H., Vale, F. A. C., & Rebelatto, J. R. (2019). Differences in timed up and go subtasks between older people with mild cognitive impairment and mild Alzheimer’s disease. Motor Control, 23(1), 1-12. https://doi.org/10.1123/mc.2017-0015 |  |
|  | Qualisys Track Manager | Qualisys | Ansai, J. H., de Andrade, L. P., Rossi, P. G., Nakagawa, T. H., Vale, F. A. C., & Rebelatto, J. R. (2019). Differences in timed up and go subtasks between older people with mild cognitive impairment and mild Alzheimer’s disease. Motor Control, 23(1), 1-12. https://doi.org/10.1123/mc.2017-0015 |  |
|  | RADAR-base passive RMT app (pRMT) | The Hyve | https://github.com/RADAR-base-Analytics/appdatacategorization |  |
|  | RADAR-base passive RMT app (pRMT) | The Hyve | Muurling, M., de Boer, C., Kozak, R., Religa, D., Koychev, I., Verheij, H., ... & Visser, P. J. (2021). Remote monitoring technologies in Alzheimer’s disease: design of the RADAR-AD study. Alzheimer's research & therapy, 13(1), 1-13. https://doi.org/10.1186/s13195-021-00825-4 |  |
|  | Rey-Osterrieth Complex Figure Test (RCFT) digital | Experimental | Kim, K. W., Lee, S. Y., Choi, J., Chin, J., Lee, B. H., Na, D. L., & Choi, J. H. (2020). A comprehensive evaluation of the process of copying a complex figure in early-and late-onset Alzheimer disease: a quantitative analysis of digital pen data. Journal of Medical Internet Research, 22(8), e18136. https://doi.org/10.2196/18136 |  |
|  | RSSI Indoor Localization System | Experimental | Masciadri, A., Comai, S., & Salice, F. (2019). Wellness assessment of Alzheimer’s patients in an instrumented health-care facility. Sensors, 19(17), 3658. https://doi.org/10.3390%2Fs19173658 |  |
|  | SENS Innovation ApS | SENS | Peimankar, A., Winther, T. S., Ebrahimi, A., & Wiil, U. K. (2023). A Machine Learning Approach for Walking Classification in Elderly People with Gait Disorders. Sensors, 23(2), 679. https://doi.org/10.3390/s23020679 |  |
|  | Sensor Platform for HEalthcare in a Residential Environment (SPHERE) | University of Bristol | Kumpik DP, Santos-Rodriguez R, Selwood J, Coulthard E, Twomey N, Craddock I, Ben-Shlomo Y. A longitudinal observational study of home-based conversations for detecting early dementia: protocol for the CUBOId TV task. BMJ open. 2022 Nov 1;12(11):e065033. https://doi.org/10.1136/bmjopen-2022-065033 |  |
|  | SensRing | BioRobotics Institute of Scuola Superiore Sant’Anna | Rovini, E., Galperti, G., Manera, V., Mancioppi, G., Fiorini, L., Gros, A., ... & Cavallo, F. (2021). A wearable ring-shaped inertial system to identify action planning impairments during reach-to-grasp sequences: a pilot study. Journal of NeuroEngineering and Rehabilitation, 18, 1-11. https://doi.org/10.1186/s12984-021-00913-4 |  |
|  | Silmee W20 | TDK Corporation | Kimura, N., Aso, Y., Yabuuchi, K., Ishibashi, M., Hori, D., Sasaki, Y., ... & Matsubara, E. (2020). Association of modifiable lifestyle factors with cortical amyloid burden and cerebral glucose metabolism in older adults with mild cognitive impairment. JAMA Network Open, 3(6), e205719-e205719. https://doi.org/10.1001/jamanetworkopen.2020.5719 |  |
|  | Smart 2-Min Mobile Alerting Method for Mild Cognitive Impairment | Experimental | Wang, Y., Chen, T., Wang, C., Ogihara, A., Ma, X., Huang, S., ... & Li, K. (2023). A New Smart 2-Min Mobile Alerting Method for Mild Cognitive Impairment Due to Alzheimer’s Disease in the Community. Brain Sciences, 13(2), 244. https://doi.org/10.3390%2Fbrainsci13020244 |  |
|  | Smart Aging Serious Game (SASG) | Consorzio di Bioingegneria e Informatica Medica | Cabinio, M., Rossetto, F., Isernia, S., Saibene, F. L., Di Cesare, M., Borgnis, F., ... & Baglio, F. (2020). The use of a virtual reality platform for the assessment of the memory decline and the hippocampal neural injury in subjects with mild cognitive impairment: The validity of smart aging serious game (SASG). Journal of clinical medicine, 9(5), 1355. https://doi.org/10.3390%2Fjcm9051355 |  |
|  | Smart Aging Serious Game (SASG) | Consorzio di Bioingegneria e Informatica Medica | Isernia, S., Cabinio, M., Di Tella, S., Pazzi, S., Vannetti, F., Gerli, F., ... & Baglio, F. (2021). Diagnostic validity of the smart aging serious game: an innovative tool for digital phenotyping of mild neurocognitive disorder. Journal of Alzheimer's Disease, 83(4), 1789-1801. https://doi.org/10.3233/jad-210347 |  |
|  | Speech Pause Analysis | Experimental | Yunusova, Y., Graham, N. L., Shellikeri, S., Phuong, K., Kulkarni, M., Rochon, E., ... & Green, J. R. (2016). Profiling speech and pausing in amyotrophic lateral sclerosis (ALS) and frontotemporal dementia (FTD). PloS one, 11(1), e0147573. https://doi.org/10.1371/journal.pone.0147573 |  |
|  | Storyteller: Automatic Story Recall Task (ASRT) | Novoic | Fristed, E., Skirrow, C., Meszaros, M., Lenain, R., Meepegama, U., Cappa, S., ... & Weston, J. (2022). A remote speech‐based AI system to screen for early Alzheimer's disease via smartphones. Alzheimer's & Dementia: Diagnosis, Assessment & Disease Monitoring, 14(1), e12366. https://doi.org/10.1002/dad2.12366 |  |
|  | Storyteller: Automatic Story Recall Task (ASRT) | Novoic | Fristed, E., Skirrow, C., Meszaros, M., Lenain, R., Meepegama, U., Papp, K. V., ... & Weston, J. (2022). Leveraging speech and artificial intelligence to screen for early Alzheimer’s disease and amyloid beta positivity. Brain Communications, 4(5), fcac231. https://doi.org/10.1093%2Fbraincomms%2Ffcac231 |  |
|  | Storyteller: Automatic Story Recall Task (ASRT) | Novoic | Skirrow, C., Meszaros, M., Meepegama, U., Lenain, R., Papp, K. V., Weston, J., & Fristed, E. (2022). validation of a Remote and Fully Automated Story Recall Task to Assess for Early Cognitive Impairment in Older Adults: Longitudinal Case-Control Observational Study. JMIR aging, 5(3), e37090. https://doi.org/10.2196/37090 |  |
|  | Storyteller: Automatic Story Recall Task (ASRT) | Novoic | Weiner, M. W., Veitch, D. P., Miller, M. J., Aisen, P. S., Albala, B., Beckett, L. A., ... & Alzheimer's Disease Neuroimaging Initiative. (2023). Increasing participant diversity in AD research: Plans for digital screening, blood testing, and a community‐engaged approach in the Alzheimer's Disease Neuroimaging Initiative 4. Alzheimer's & Dementia, 19(1), 307-317. https://doi.org/10.1002/alz.12797 |  |
|  | Stricker Learning Span (SLS) | Experimental | Stricker, N. H., Stricker, J. L., Karstens, A. J., Geske, J. R., Fields, J. A., Hassenstab, J., ... & Mielke, M. M. (2022). A novel computer adaptive word list memory test optimized for remote assessment: Psychometric properties and associations with neurodegenerative biomarkers in older women without dementia. Alzheimer's & Dementia: Diagnosis, Assessment & Disease Monitoring, 14(1), e12299. https://doi.org/10.1002/dad2.12299 |  |
|  | TEMPO (technology-enabled medical precision observation) nodes | University of Virginia Engineering | Rose, K. M., Lach, J., Perkhounkova, Y., Gong, J., Dandu, S. R., Dickerson, R., ... & Stankovic, J. (2018). Use of body sensors to examine nocturnal agitation, sleep, and urinary incontinence in individuals with Alzheimer's disease. Journal of Gerontological Nursing, 44(8), 19-26. https://doi.org/10.3928/00989134-20180626-03 |  |
|  | Tobii eye tracker | Tobii | Bott, N., Madero, E. N., Glenn, J., Lange, A., Anderson, J., Newton, D., ... & Zola, S. (2018). Device-embedded cameras for eye tracking–based cognitive assessment: validation with paper-pencil and computerized cognitive composites. Journal of medical Internet research, 20(7), e11143. https://doi.org/10.2196/11143 |  |
|  | Tobii eye tracker | Tobii | Li, N., Yang, X., Du, W., Ogihara, A., Zhou, S., Ma, X., ... & Li, K. (2022). Exploratory Research on Key Technology of Human-Computer Interactive 2.5-Minute Fast Digital Early Warning for Mild Cognitive Impairment. Computational Intelligence and Neuroscience, 2022. https://doi.org/10.1155/2022/2495330 |  |
|  | Traffic Light for Dementia” (TLiDe) | Experimental | Kwon, S. J., Kim, H. S., Han, J. H., Bae, J. B., Han, J. W., & Kim, K. W. (2021). Reliability and Validity of Alzheimer's Disease Screening With a Semi-automated Smartphone Application Using Verbal Fluency. Frontiers in Neurology, 12, 684902. https://doi.org/10.3389/fneur.2021.684902 |  |
|  | VALMA (Voice, Activity, and Location Monitoring for Alzheimer’s Disease) | Neural & Rehabilitation Lab, University of Waterloo | Tung, J. Y., Rose, R. V., Gammada, E., Lam, I., Roy, E. A., Black, S. E., & Poupart, P. (2014). Measuring life space in older adults with mild-to-moderate Alzheimer's disease using mobile phone GPS. Gerontology, 60(2), 154-162. https://doi.org/10.1159/000355669 |  |
|  | Vehicular Onboard Data System | University of Michigan Transportation Research Institute | Eby, D. W., Silverstein, N. M., Molnar, L. J., LeBlanc, D., & Adler, G. (2012). Driving behaviors in early stage dementia: A study using in-vehicle technology. Accident Analysis & Prevention, 49, 330-337. https://doi.org/10.1016/j.aap.2011.11.021 |  |
|  | Verily Study Watch | Verily | Chen, C., Kowahl, N. R., Rainaldi, E., Burq, M., Munsie, L. M., Battioui, C., ... & Kapur, R. (2023). Wrist-worn sensor-based measurements for drug effect detection with small samples in people with Lewy Body Dementia. Parkinsonism & Related Disorders, 109, 105355. https://doi.org/10.1016/j.parkreldis.2023.105355 |  |
|  | Versatile wireless EEG | Bitbrain | Perez-Valero, E., Lopez-Gordo, M. Á., Gutiérrez, C. M., Carrera-Muñoz, I., & Vílchez-Carrillo, R. M. (2022). A self-driven approach for multi-class discrimination in Alzheimer's disease based on wearable EEG. Computer Methods and Programs in Biomedicine, 220, 106841. https://doi.org/10.1016/j.cmpb.2022.106841 |  |
|  | Vestibular Rotation Task | Experimental | Coughlan, G., Plumb, W., Zhukovsky, P., Aung, M. H., & Hornberger, M. (2023). Vestibular contribution to path integration deficits in ‘at-genetic-risk’for Alzheimer’s disease. Plos one, 18(1), e0278239. https://doi.org/10.1371/journal.pone.0278239 |  |
|  | Vicon Nexus 2.8 | Vicon | Ali, N., Liu, J., Tian, H., Pan, W., Tang, Y., Zhong, Q., ... & Zhu, Y. (2022). A novel dual-task paradigm with story recall shows significant differences in the gait kinematics in older adults with cognitive impairment: A cross-sectional study. Frontiers in Aging Neuroscience, 14. https://doi.org/10.3389/fnagi.2022.992873 |  |
|  | ViewMind | ViewMind | Parra, M. A., Granada, J., & Fernández, G. (2022). Memory‐driven eye movements prospectively predict dementia in people at risk of Alzheimer's disease. Alzheimer's & Dementia: Diagnosis, Assessment & Disease Monitoring, 14(1), e12386. https://doi.org/10.1002/dad2.12386 |  |
|  | Virtual Supermarket Test (VST) | Centre for Research and Technology Hellas/ Information Technologies Institute (CERTH/ITI) in collaboration with the Greek Association of Alzheimer’s Disease and Related Disorders (GAADRD) | Iliadou, P., Paliokas, I., Zygouris, S., Lazarou, E., Votis, K., Tzovaras, D., & Tsolaki, M. (2021). A comparison of traditional and serious game-based digital markers of cognition in older adults with mild cognitive impairment and healthy controls. Journal of Alzheimer's disease, 79(4), 1747-1759. https://doi.org/10.3233/jad-201300 |  |
|  | Virtual Supermarket Test (VST) | Centre for Research and Technology Hellas/ Information Technologies Institute (CERTH/ITI) in collaboration with the Greek Association of Alzheimer’s Disease and Related Disorders (GAADRD) | Zygouris, S., Ntovas, K., Giakoumis, D., Votis, K., Doumpoulakis, S., Segkouli, S., ... & Tsolaki, M. (2017). A preliminary study on the feasibility of using a virtual reality cognitive training application for remote detection of mild cognitive impairment. Journal of Alzheimer's Disease, 56(2), 619-627. https://doi.org/10.3233/jad-160518 |  |
|  | VisMET (mobile) | Experimental | Haque, R. U., Pongos, A. L., Manzanares, C. M., Lah, J. J., Levey, A. I., & Clifford, G. D. (2020). Deep convolutional neural networks and transfer learning for measuring cognitive impairment using eye-tracking in a distributed tablet-based environment. IEEE Transactions on Biomedical Engineering, 68(1), 11-18. https://doi.org/10.1109/tbme.2020.2990734 |  |
|  | W180 | Shenzhen Fitfaith Technology | Liu, Z., Zhang, L., Wu, J., Zheng, Z., Gao, J., Lin, Y., ... & Zhou, Y. (2022). Machine learning-based classification of circadian rhythm characteristics for mild cognitive impairment in the elderly. Frontiers in Public Health, 10, 1036886. https://doi.org/10.3389%2Ffpubh.2022.1036886 |  |
|  | Winterlight Labs Speech Assessment | Winterlight Labs / Cambridge Cognition | Balagopalan, A., Eyre, B., Robin, J., Rudzicz, F., & Novikova, J. (2021). Comparing pre-trained and feature-based models for prediction of Alzheimer's disease based on speech. Frontiers in aging neuroscience, 13, 635945. https://doi.org/10.3389/fnagi.2021.635945 |  |
|  | Winterlight Labs Speech Assessment | Winterlight Labs / Cambridge Cognition | Fraser, K. C., Meltzer, J. A., & Rudzicz, F. (2016). Linguistic features identify Alzheimer’s disease in narrative speech. Journal of Alzheimer's Disease, 49(2), 407-422. https://doi.org/10.3233/jad-150520 |  |
|  | Winterlight Labs Speech Assessment | Winterlight Labs / Cambridge Cognition | Fraser, K. C., Meltzer, J. A., Graham, N. L., Leonard, C., Hirst, G., Black, S. E., & Rochon, E. (2014). Automated classification of primary progressive aphasia subtypes from narrative speech transcripts. cortex, 55, 43-60. https://doi.org/10.1016/j.cortex.2012.12.006 |  |
|  | Winterlight Labs Speech Assessment | Winterlight Labs / Cambridge Cognition | Fraser, K. C., Rudzicz, F., & Rochon, E. (2013, August). Using text and acoustic features to diagnose progressive aphasia and its subtypes. In Interspeech (pp. 2177-2181). http://dx.doi.org/10.21437/Interspeech.2013-514 |  |
|  | Winterlight Labs Speech Assessment | Winterlight Labs / Cambridge Cognition | Kindellan, R., Sirotkin, S., Xu., M., Fidalgo, C., Simpson, W., & Robin, J. (2022, Nov 29-Dec 2). Accuracy of automated scoring of word recall assessments. [Poster presentation.] Clinical Trials for Alzheimer’s Disease (CTAD), San Francisco, CA, USA. Winterlight Labs, Toronto, ON, Canada. |  |
|  | Winterlight Labs Speech Assessment | Winterlight Labs / Cambridge Cognition | Kindellan, R., Xu, M., Ruan, J., Sirotkin, S., Robin, S. (2022). Analytical validation of automatic speech recognition tools used for voice biomarker development. [Poster presentation.] ISCTM Autumn Conference, 2022. Winterlight Labs, Toronto, ON, Canada. |  |
|  | Winterlight Labs Speech Assessment | Winterlight Labs / Cambridge Cognition | Robin, J., Xu, M., Detke, M., & Simpson, W. (2022, Nov 29 - Dec 2). validation of an objective, speech-based object content score for measuring disease progression in AD. [Poster presentation.] Winterlight Labs, Toronto, ON, Canada. Detke Biopharma Consulting, Carmel, IN, USA. Department of Psychiatry and Behavioural Neuroscience. McMaster University, Hamilton, ON, Canada, |  |
|  | Winterlight Labs Speech Assessment | Winterlight Labs / Cambridge Cognition | Robin, J., Xu, M., Kaufman, L. D., & Simpson, W. (2021). Using digital speech assessments to detect early signs of cognitive impairment. Frontiers in digital health, 3, 749758. https://doi.org/10.3389/fdgth.2021.749758 |  |
|  | Winterlight Labs Speech Assessment | Winterlight Labs / Cambridge Cognition | Sorinas, J., Robin, J., Simpson, W., Curcic, J., & Hannesdottir, K. (2023). Speech phenotypes in cognitively healthy participants at risk of developing Alzheimer’s disease. [Poster presentation.] Novartis Institutes for Biomedical Research, Basel, Switzerland. Winterlight Labs, Inc. Toronto, Canada. McMaster University, Hamilton, Canada. Novartis Institutes for Biomedical Research, Cambridge, MA, United States. |  |
|  | Winterlight Labs Speech Assessment | Winterlight Labs / Cambridge Cognition | Yancheva, M., Fraser, K. C., & Rudzicz, F. (2015, September). Using linguistic features longitudinally to predict clinical scores for Alzheimer’s disease and related dementias. In Proceedings of SLPAT 2015: 6th Workshop on Speech and Language Processing for Assistive Technologies (pp. 134-139). https://doi.org/10.18653/v1/W15-5123 |  |
|  | Winterlight Labs Speech Assessment | Winterlight Labs / Cambridge Cognition | Yeung, A., Iaboni, A., Rochon, E., Lavoie, M., Santiago, C., Yancheva, M., ... & Mostafa, F. (2021). Correlating natural language processing and automated speech analysis with clinician assessment to quantify speech-language changes in mild cognitive impairment and Alzheimer’s dementia. Alzheimer's research & therapy, 13(1), 109. https://doi.org/10.1186/s13195-021-00848-x |  |
|  | Withings Go | Withings | McWilliams, E. C., Barbey, F. M., Dyer, J. F., Islam, M. N., McGuinness, B., Murphy, B., ... & Buick, A. R. (2021). Feasibility of repeated assessment of cognitive function in older adults using a wireless, mobile, dry-EEG headset and tablet-based games. Frontiers in psychiatry, 12, 574482. https://doi.org/10.3389/fpsyt.2021.574482 |  |
|  | Zeno Walkway | Protokinetics | Pieruccini-Faria, F., Sarquis-Adamson, Y., & Montero-Odasso, M. (2019). Mild cognitive impairment affects obstacle negotiation in older adults: results from “gait and brain study”. Gerontology, 65(2), 164-173. https://doi.org/10.1159/000492931 |  |
